# Validation of the online version of the sharenting evaluation scale (SES) in Iranian parents: Psychometric properties and concurrent validity

**DOI:** 10.1002/brb3.3300

**Published:** 2023-11-05

**Authors:** Faezeh Peimanpak, Abbas Abdollahi, Kelly A. Allen, Feruza Abulkosimovna Rakhmatova, Alaa Aladini, Shadia Hamoud Alshahrani, Jocelyn Brewer

**Affiliations:** ^1^ Department of Counseling, Faculty of Education and Psychology Alzahra University Tehran Iran; ^2^ Educational Psychology and Inclusive Education, Faculty of Education Monash University Melbourne Australia; ^3^ Department of Educational Theory of Pedagogy Jizzakh State Pedagogical University Jizzakh Uzbekistan; ^4^ Department of Education, Faculty of Arts and Applied Sciences Dhofar University Salalah Oman; ^5^ Department of Nursing, Faculty of Medical Surgical Nursing King Khalid University Khamis Mushait Saudi Arabia; ^6^ Cyberpsychology Research Group University of Sydney Camperdown Australia

**Keywords:** parents, sharenting, social comparison, social well being

## Abstract

**Introduction:**

This study examines the psychometric properties of the online version of the sharenting evaluation scale (SES) with a sample of Iranian parents.

**Methods:**

A sample population of 240 parents (25% fathers and 75% mothers) with an average age of 39.63 (standard deviation = 10.41) were selected by convenience sampling method and completed the Persian version of the SES online.

**Results:**

Findings showed that concurrent validity of the SES with social comparison and social well being was within the acceptable range. The Cronbach's alpha coefficient for the SES was 0.92, and for the subscales of self‐control, social behavior, and implications were, respectively, 0.84, 0.71, and 0.70, which indicated good internal consistency between the items. Confirmatory factor analysis supported the Persian version of this scale same as the English version. The Persian version of the SES demonstrated psychometric properties of validity and reliability within the acceptable range (*α* = .70 and .84). In the present study, the results of convergent validity showed that there is a positive and significant relationship between sharing and constructs, such as social comparison, self‐control, implications, and social behaviors, but no significant relationship was found between social well being and sharing.

**Conclusions:**

Persian version of the SES is a suitable scale to measure the degree to which parents share their children's sensitive content on the Internet and social media.

## Graphical abstract

• Parents of young children actively use social media networks and often share their children's activities and accomplishments with enthusiasm through images and subtitles on these platforms. • The sharenting evaluation scale (SES) measures the rate of sharing children's characteristics and its reasons. • This psychometric study is about the SES among Iranian parents. • This scale has acceptable psychometric properties to assess sharing the information about children among parents.

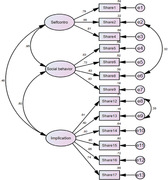

## 1 INTRODUCTION

Social issues generally refer to common problems that affect people within a community. However, as the result of changing cultural, economic, or social circumstances, single issues such as “sharenting” can develop into a larger phenomenon involving many people. Sharenting is often the cause of conflicting opinions about what is perceived as correct or incorrect behavior in personal or interpersonal social life (Ferrara et al., 2023). Parents of young children are active users of social media networks (Cino, 2022) and are often enthusiastically engaged in sharing their children's activities and achievements via images and captions on these platforms (Blum‐Ross & Livingstone, 2017). The term “sharenting,” a portmanteau of “share” and “parenting,” is commonly used to describe the digital narrations of family life, most commonly of younger children (Romero‐Rodríguez et al., 2022). Sharenting often commences in utero, with a pregnancy announcement and post of the sonogram of the unborn child a commonplace practice (Leaver, 2017), signaling the commencement of various sharenting decisions and practices, which see on average 1000 photos of a child being shared by the time they are 5‐year old (Plunkett, 2019).

Sharenting has positive aspects for parents. Several researchers have noted benefits that can be obtained through sharenting practices (Ong et al., 2022; Romero‐Rodríguez et al., 2022). Online platforms provide a range of benefits to parents as they both seek and provide support and advice, social connectedness, and seek to feel less alone in the complexity of parenting (Ouvrein & Verswijvel, 2019).

Some parents frequently share volumes of content in a way that seeks to provoke certain responses, often related to affirming their child's behavior or attractiveness, or attracting comments encouraging and confirming their position as a “good parent.” It is mothers who are more likely to engage in sharenting practices (Cataldo et al., 2022) and seek support and connection through, capturing, documenting, captioning, and posting content. But parents’ decisions to use social media matter, not only for the parents. Since posts last forever—because the Internet is archived those decisions have long‐term implications for their children (Williams‐Ceci et al., 2021).

Most parents continue to share information and pictures of their children without any precautions, often due to a false sense of security about the privacy settings (Ferrara et al., 2023). However, the same authors warn about the “dark side” of sharenting: The emergence of computerized childhood, loss of privacy, and the distress it could cause children in the future. Recent statistics have revealed its social dimension and potential risks. Identity theft is one of the major risks associated with sharenting. Various scholars and social commentators (Sarkadi et al., 2020) have argued that the most concerning aspect of sharenting is that parents are sharing aspects of their children's identities without their consent and debate the ability of an infant or toddler to provide this. The prevalence of sharenting has caused some researchers to call it *parental oversharing* or extreme sharing (Klucarova & Hasford, 2021).

Parents may inadvertently share sensitive information about their child's identity (date and place of birth), health status, and the school they attend (when photos fail to obscure the uniform or school signage), which may place children at risk of identity theft and potential physical danger. Privacy concerns and the implication of these in relation to sharenting practices remain a significant feature of the research literature (Ní Bhroin et al., 2022; Ouvrein & Verswijvel, 2019; Siibak & Traks, 2019).

The prevalence of sharenting has caused some researchers to call it *parental oversharing* or extreme sharing (Klucarova & Hasford, 2021). The seeking of affirmation and positive reinforcement in sharenting is also a negative feature of the practice which has been investigated (Ong et al., 2022; Ouvrein & Verswijvel, 2019). In sum, disclosing the child's personal information on social media affects the child's privacy. Sometimes, the child has no control over the content parents share online, let alone over the possibility of allowing their parents to post any information or to create their digital narrative (McTigue, 2021; Ranzini et al., 2020; Steinberg, 2017).

In sum, sharenting syndrome is common among parents. The behaviors of sharing and disclosing intimate information about children by their parents on social media platforms are rapidly growing and have become a topic of research for scholars worldwide (Doğan Keskin et al., 2023). Although social media has undoubtedly provided a space for parents to share experiences and receive support around parenting, sharenting remains a contestable issue. Thus, one reading of sharenting would be as a display of good parenting as mothers “show off” their children as a marker of success. However, the term also can be used pejoratively to describe parental oversharing of child‐focused images and content (Lazard et al., 2019). Sharenting is a recent phenomenon in which parents disclose detailed information about their children online, which can risk their children's long‐term safety and parental relationships. To mitigate these risks and discourage the sharing of inappropriate content, educating parents and caregivers across all stages of their parenting journey to be aware of and have strategies to manage a range of cyber psychological and cyber safety issues will help avert some of the negative privacy and data implications of sharenting habits (Cataldo et al., 2022; Williams‐Ceci et al., 2021). They can also be helped to balance their natural inclination to share pride in their children's progress and educated about.

However, interventions in this field require further studies with valid assessment tools. A measurement tool has been developed that can be used to determine the prevalence of sharenting syndrome. Sharenting evaluation scale (SES) assesses the degree of sharenting performed by an adult (Romero‐Rodríguez et al., 2022). However, no studies have determined the validity and reliability of the Persian version of the SES and no psychometric properties have been available to Iranian mental health researchers so far. Based on these considerations and due to the nonexistence of a scale that assesses sharenting behavior in an Iranian population, the aim of the current study is to validate the Romero‐Rodríguez et al. (2022) SES in Persian to broaden the scale's use and utility to the Iranian population.

## 2 THE PRESENT STUDY

The SES is the only empirical measure of sharenting currently available. It has been validated with populations in Spain (Kopecky et al., 2020; Romero‐Rodríguez et al., 2022) and used in Malaysia (REF), and while yielding promising results as an empirical measure of sharenting, it has not been validated for use with parents in Iran. In the Spanish evaluation, Romero‐Rodríguez et al. (2022) confirmed that the 17 items of this scale held robust psychometric properties across three factors: self‐control, social behavior, and implications. Given the potential negative implications of sharenting and the absence of a measure to assess sharenting in an Iranian population, this study examined the psychometric properties of the SES using online platforms in a sample of Iranian parents.

## 3 METHODOLOGY

### 3.1 The participants

Participants in the study were 240 parents (60 male = 25% and 180 female = 75%) aged between 18 and 72 (average age = 39.63 and standard deviation = 10.41). All participants were parents. Sixty‐three parents (26.3%) reported having one child; 56 (23.3%), two children, 97 (40.4%), three children; 17 (7.1%), four children; 3 (1.3%), five children, and one parent (0.4%) reported having six children. The parent participants in the study identified as having various levels of educational attainment; 7 parents (2.9%) were middle school graduates, 32 (13.3%) were high school graduates, 106 (44.2%) were college graduates, 80 (33.3%) had Master's degrees, and 14 (5.8%) parents had Doctoral degrees. Participants were selected using a convenience sampling method. The Inclusion criterion was Persian as a native language and familiarity with virtual platforms, and exclusion criteria were “having a severe psychiatric disorder and unwillingness to participate in research.”

All of the two hundred and forty parents completed the questionnaires. Mahalanobis Distance (D2) square was less than 4 (Tabachnick and ve Fidell, 2012). No outliers were observed in the data.

### 3.2 Procedure

All participants provided their informed consent prior to completing the 15‐min online questionnaire created in Google Forms. This questionnaire collected participants’ demographic data, including age, gender, and education. Data collection occurred between April 2022 and June 2022 in accordance with test administration regulations of Alzahra University Research Ethics Committee (IR/11/28/1401).

All measures (i.e., the SES, The Social Well‐being Questionnaire, (Keyes, 1998); and the social comparison scale, (Gibbons & Buunk, 1999)) were translated into Persian, and their validity and reliability confirmed in past research (REF). The Back Translation technique was used to translate the English versions of the measures. First, two native Persian translators translated the English version of SES to Persian. They compared and contrasted their translated scripts to ensure no inconsistencies between the two texts. In the next stage, an expert in Persian Literature edited the revised English to Persian translation. The edited version of SES was then referred to two native English translators to determine that the text was translated accurately. In the following stage, an English expert retranslated the text to English. In the final stage, the original and retranslated versions of SES were referred to a third translator who was an expert in both English and Persian language. At this stage, the Persian translation of SES was finally approved. After approving the final draft of the Persian version of SES and resolving the ambiguities within the questionnaire, it was administered to 30 parents as a trial to detect and resolve additional issues with the comprehension of questionnaire items. Participants were not offered financial compensation for participating in the study.

### 3.3 Primary data analysis: face validity

Prior to data analysis, the test validity of the Persian version of SES was checked. First, the face validity of the scale was examined. Experts reached a consensus that the items in the Persian version of SES accurately reflected the target construct (Sharenting). The face validity was examined using two methods. The first method, using the proposed method by Boateng et al. (2018), the qualitative aspects of SES were examined (Boateng et al., 2018). A total of 15 test administrators that were not experts in Persian were interviewed by phone. Each of these test administrators was asked whether they believed that the items within the Persian version of SES reflected upon the aspects of sharing videos and images online or not. The face validity was analyzed using a quantitative approach in the second method. The quantitative approach provided an excellent measure to examine the questionnaire items based on their internal consistency, difficulty, and ambiguity to see whether they provide an appropriate medium to measure sharenting or not (Hajizadeh & Asghari, 2011). The SES face validity was analyzed using a quantitative approach and administering the test to 15 participants to determine the scale's impact score (impact score = frequency (%) × importance) (Rose et al., 2009). *Importance* is the rate at which the participants believe a specific item expounds on a specific construct and indicates the *frequency* of specific answers.

### 3.4 Primary data analysis: content validity

The content validity of the SES was also examined using the following two approaches: quantitative and qualitative. The same 15 test administrators familiar with the qualitative approach and existing literature on sharenting examined all 17 items of SES. To provide a quantitative analysis of the content validity, the content validity index (I‐CVI) and content validity ratio (I‐CVR) were calculated (Cook & Beckman, 2006). By pointing out one of the items on the Likert Scale of 1 (very seldom or not true of me) and 4 (very often true or true of me), the content validity was determined by dividing the number of the experts who chose items 3 and 4 into the number of all the experts who filled the questionnaire. Content validity provides an expert analysis of the degree to which the test items evaluate all aspects of the construct designed to measure. To determine the content validity of the SES items, the experts were asked to decide the necessity of each item by choosing items 1 (“it is not necessary”) and 3 (“it is necessary”). The content validity was determined by subtracting the number of all the experts who considered one item as necessary from the number of all the experts who took part in this content analysis and dividing the result into the number of all the experts who took part in this survey. According to Polit et al. (2007), the I‐CVI of 0.7 and higher indicates an appropriate level of content validity (Polit et al., 2007). Moreover, for the 15 experts who took part in this survey, the content validity ratio (I‐CVR) of 0.49 and higher indicates an appropriate level of content validity (Lawshe, 1975). Therefore, the scale content validity index (S‐CVI) was determined by counting the number of items on a scale deemed “highly relevant.” The S‐CVI can be determined using the following methods: The Universal Agreement and The scale content validity index/average (S‐CVI/Ave); the second method is a more conservative approach. “S‐CVI/UA is calculated by adding all items with I‐CVI equal to 1 divided by the total number of items, while S‐CVI/Ave is calculated by taking the sum of the I‐CVIs divided by the total number of items. An S‐CVI/Ave ≥0.9 has excellent content validity.” (Shi et al., 2012)

### 3.5 Proper data analysis: construct validity

To determine whether or not the Persian version of SES supports the tri‐factor construct similar to Romero‐Rodríguez's (2022) scale, SPSS 26 and AMOS‐24 were employed for data analysis and descriptive statistics. Confirmatory factor analysis was used to examine the scale's factor structure. According to approach, negative or lower than 0.40‐factor loading is deemed not acceptable in this study. Moreover, the average variance extracted (AVE) was employed to determine the convergent validity of the scale. A 0.4 or higher AVE is within the acceptable range in this study (Henseler et al., 2015). Construct reliability (CR), and Cronbach's alpha determined the scale's internal consistency. A 0.7 or higher reliability is within the acceptable range on this scale (Elvén et al., 2018), whereas a 0.07 or higher Cronbach's alpha is within the acceptable range to determine the internal consistency in social science (Taber, 2018).

### 3.6 Proper data analysis: concurrent validity

To determine the concurrent validity of SES (Romero‐Rodríguez et al., 2022), the Pearson correlation coefficient, the mental health continuum (Keyes, 1998), and the social comparison scale (Gibbons & Buunk, 1999) were employed. Therefore, we examined the concurrent validity of the Persian version of SES by conducting statistical analysis similar to the mental health continuum (Keyes, 1998), and the social comparison scale (Gibbons & Buunk, 1999).

### 3.7 Measures

*Social well‐being scales* (Keyes, 1998): This scale consists of 20 items organized into 5 dimensions: social integration, social acceptance, social contribution, social actualization, and social coherence (Keyes, 1998). Through a Likert scale from 1 to 7 (from strongly disagree to strongly agree), participants are asked to rate their degree of social well being. Higher scores mean that socially healthier people should not see society as unpleasant and see themselves as important members. They should care about and feel safe in the community, living a coherent life.

*Sharenting evaluation scale* (SES) (Romero‐Rodríguez et al., 2022): This scale consists of 17 items organized in 3 dimensions: Implications (composed of items 11, 12, 13, 14, 15, 16, and 17), social behavior (composed of items 5, 6, 7, 8, 9, and 10,), and self‐control (composed of 4 items 1, 2, 3, and 4). Through a Likert scale from 0 to 5 (from never to always), the overall reliability of the instrument was acceptable (*α* = .76). On the other hand, for each of the dimensions, the reliability was as follows: implications (*α* = .871); social behavior (*α* = .697); self‐control (*α* = .672).

The face validity results indicated that impact scores for all items of SES were higher than 1.5. Therefore, SES has strong face validity (see Table 1). CVI results indicated a score of 0.79 and higher for all SES items indicating a strong CVI. CVR results indicated a CVR score higher than the minimum defined by Lawshe (1975) table, indicating that all items incorporated in this scale were necessary to examine the target construct of sharenting (see Table 2).

**TABLE 1:** Impact scores for the items of sharenting evaluation scale (SES) scale.

Item		Difficulty	Relevancy	Ambiguity
1	How often have you shared pictures or videos of the minor on your social media profile?	2.87	2.66	3.36
2	How often have you sent photographs or videos of the minor by private message to another person?	2.28	2.16	2.34
3	How often have you shared more than one photo or video per day?	2.16	2.22	2.28
4	How often have you felt the need to want to share the minor's photographs or videos on social media?	2.28	2.28	2.16
5	How often have you shared a photo or video of the minor in order to receive positive feedback from your contacts?	2.22	2.28	2.8
6	How often have you shared photographs or videos of the minor in intimate situations (e.g., nude or semi‐nude, in swimwear or in situations where sensitive information is exposed)?	2.28	2.73	2.28
7	How often have you shared photographs or videos that may cause frustration and/or embarrassment to the minor?	2.22	2.73	2.66
8	How often have you shared pictures or videos of other minors that you have received from other people (e.g., pictures of children of a family member or friend or even memes, stickers or viral videos)?	2.4	2.28	2.22
9	How often have people around you reproached you for sharing photos or videos of the minor?	2.22	2.73	1.85
10	How often have you deleted the photo or video after sharing it on social media after receiving feedback from someone else?	2.73	2.22	2.66
11	How often have you felt that you were invading the minor's privacy by sharing the child's photograph or video?	2.8	2.8	2.28
12	How often have you considered the Child Protection Act when sharing your photo or video?	2.22	2.16	2.66
13	How often have you considered that the photographs or videos you share on social media are creating a digital footprint of the minor?	1.9	2.28	2.28
14	How often have you considered that the photograph or video shared may have a negative impact on the minor's future?	2.73	2.66	2.73
15	How often have you considered that sharing a photo or video presents a risk to the minor?	2.22	2.22	2.1
16	How often have you considered that the photographs or videos you have shared of the minor could be used for identity theft on the Internet?	2.16	2.28	2.28
17	How often have you considered that the photographs or videos you have shared of the minor could end up on websites that promote pedophilia?	1.75	1.8	1.8

**TABLE 2:** Content validity index (CVI) and content validity ratio (CVR) values for the items of sharenting evaluation scale (SES) scale.

	CVI		CVR			Corrected item‐total correlation	if the item is deleted		
Item	Difficulty	Relevancy	Ambiguity	Urgency	M (SD)			Skewness	Kurtosis
1	1	0.93	0.86	0.73	1.06 (1.07)	0.52	0.81	0.75	−0.07
2	0.93	0.86	1	0.6	1.42 (0.99)	0.41	0.82	0.47	0.21
3	0.86	0.8	0.86	0.86	1.91 (1.18)	0.26	0.82	0.27	−0.39
4	0.8	0.86	0.86	0.6	1.10 (1.06)	0.51	0.81	0.9	1.02
5	1	0.93	0.86	0.73	0.86 (0.97)	0.56	0.81	0.81	0.1
6	0.93	0.86	0.93	0.73	0.38 (0.69)	0.58	0.81	1.7	1.82
7	0.86	0.8	0.86	0.6	0.55 (0.92)	−0.018	0.83	2.08	5.21
8	0.93	0.86	0.93	0.73	0.98 (0.99)	0.41	0.82	0.73	0.09
9	0.8	0.93	0.86	0.6	0.34 (0.64)	0.27	0.86	1.77	2.13
10	1	0.86	0.86	0.86	4.10 (1.09)	−0.14	0.84	−1.55	2.99
11	0.86	0.8	0.86	0.73	4.55 (0.99)	0.16	0.83	−2.89	9.12
12	0.93	0.93	0.93	0.6	2.40 (1.96)	0.59	0.8	0.06	−1.57
13	0.86	0.86	0.8	0.73	3.80 (1.58)	0.38	0.82	−1.21	0.26
14	0.93	0.8	0.93	0.73	2.84 (1.87)	0.67	0.8	−0.35	−1.37
15	0.86	0.86	0.86	0.6	2.92 (1.86)	0.7	0.79	−0.38	−1.31
16	0.8	0.93	0.86	0.73	2.90 (1.84)	0.69	0.79	−0.38	−1.27
17	0.86	0.8	0.8	0.6	3.34 (1.89)	0.64	0.8	−0.73	−1.02

Table 2 demonstrates that CVR and CVI are higher than what was already observed, indicating a strong content validity. Moreover, within the table, the Pearson correlation and Cronbach's alpha coefficient for all items, except for items 3, 7, 10, and 11 that were deleted due to their correlation coefficient of lower than 0.3, were reported at acceptable ranges. Table 2 demonstrates that the normality testing done by skewness and kurtosis indicates a normal distribution for all SES items, except for items 7 and 11.

The acceptable cut‐off scores for measurement fit indices are CMIN/DF (chi‐square/degree of freedom) <5; CFI (comparative fit index) >0.90; RMSEA (root mean square error of approximation) <0.08; TLI (Tucker‐Lewis index) >0.90; and GFI (Goodness of fit index) >0.90 (Abd‐El‐Fattah, 2010). The findings revealed the model had appropriate measurement fit indices (CMIN/DF = 1.730, CFI = 0.97, RMSEA = 0.05, TLI = 0.96, GFI = 0.94, IFI = 0.97, NFI = 0.93, RFI = 0.91).

**FIGURE 1:**
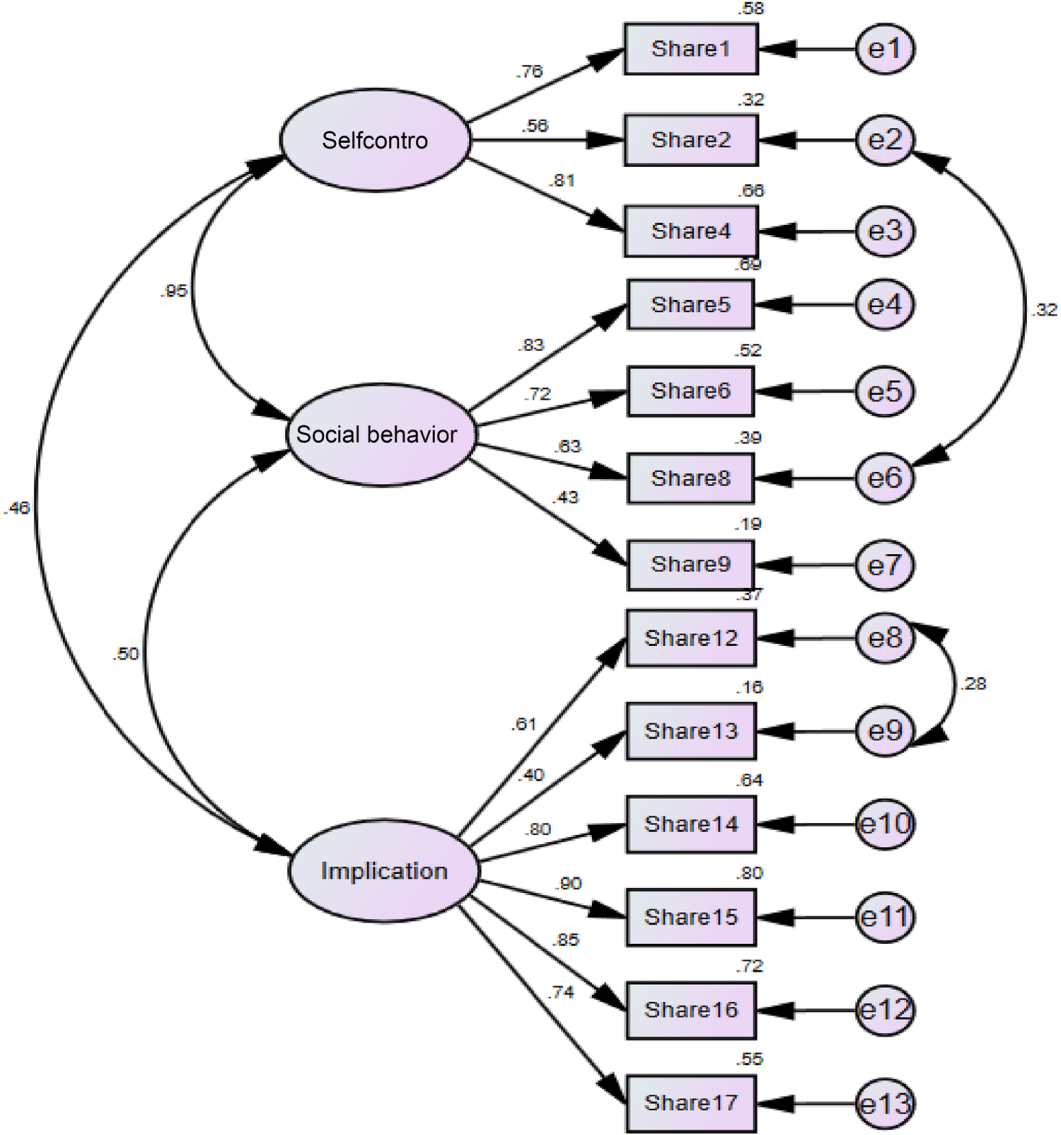
Confirmatory factor analysis estimates. *Note*: *χ* ^2^ = 103.772; df = 60; *p*‐value = .05.

All scales used to determine the underlying measures of fit demonstrated proper goodness of fit, indicating that the tri‐factor model, by deleting items 3, 7, 10, and 11 that had a factor loading of less than 0.40, suitably represents the data in the actual population (see Figure 1). Moreover, as demonstrated in Figure 1, all items demonstrated a sufficient factor loading on the latent factor (which is statistically significant between 0.4 and 0.9 with *p* ≥ .001), indicating higher support for item distribution within the spectrum of the latent variable. Furthermore, because Kline's approach (Kline, 2016) indicated a factor loading of higher than 0.40 for all 13 items and the goodness of fit of the proposed model, no corrective measures that contained any lateral loading were enforced on the test. Therefore, each item represented a unique criterion of the latent variable in question (sharenting evaluation).

The overall reliability of the instrument was acceptable (*α* = .864). On the other hand, for each of the dimensions the reliability was as follows: implications (*α* = .870); social behavior (*α* = .735); self‐control (*α* = .758).

AVE for “self‐control” (AVE = 0.52), “social behavior” (AVE = 0.44), “implications” (AVE = 0.54), and for the entire scale demonstrated an acceptable convergent validity (AVE = 0.506). In contrast, CR was reported at 0.79, which was higher than the proposed 0.7 by Tabachnick and ve Fidell (2012) (CR > AVE), indicating a good internal consistency between the SES items. Finally, Cronbach's alpha coefficient (*α*) for the entire scale was reported at 0.86, indicating a good internal consistency between the SES items (Taber, 2018).

As shown in Table 3, the Pearson correlation coefficient results indicated a positive and significant correlation between the social comparison scale and SES (*r* = .33, *p* < .001). There is a negative and significant correlation between social comparison and the mental health continuum (*r* = −.13, *p* < .05). There was no correlation between the mental health continuum and SES. There is a positive and significant correlation between each of the following factors, including “self‐control” (*r* = .67, *p* < .001), “implication” (*r* = .92, *p* < .001), and “social behavior” (*r* = .69, *p* < .001) and SES. Therefore, the results of this study indicate that the Persian version of SES is a valid and reliable research tool for adults.

**TABLE 3:** Correlations between the studied variables.

Variables	M	SD	1	2	3	4	5	6
(1) Social comparison	47.51	12.36						
(2) Social well being	68.29	6.79	−0.13					
(3) Self‐control	3.57	2.57	0.37	−0.066				
(4) Implications	18.2	8.6	0.19	−0.6	0.37			
(5) Social behaviors	2.55	2.51	0.45	−0.032	0.76	0.4		
(6) Sharenting	24.33	11.44	0.33	−0.067	0.67	0.92	0.69	1

## 4 DISCUSSION

This study examined the psychometric properties of Spanish SES, the factor structure, and validity to determine whether or not it can be applied to an Iranian sample. A confirmatory factor analysis supported the SES tri‐factor model by deleting items 3, 7, 10, and 11. The study's findings fall in line with the findings of Romero‐Rodríguez et al. (2022), Kopecky et al. (2020), Verswijvel et al. (2019), and (Verswijvel et al., 2019).

The present study found that the Spanish SES is a valuable scale to examine sharenting within Persian culture based on the psychometric properties of the Persian version of SES on a sample of Iranian parents. When it comes to sharenting, a central concern is maintaining a child's privacy. Since parents’ access to social media platforms is increasing by the day in Iran, the validity of the Persian SES has implications for future studies on sharenting in Iran. The SES will allow researchers to better identify parental behavior on social media platforms in order to design and implement precautionary and educational efforts to help parents navigate social media platforms in Iran safely (Kopecky et al., 2020).

The Back translation method (English to Persian and Persian to English) used in this study indicates that SES (Romero‐Rodríguez et al., 2022) is translated to Persian correctly. Moreover, the Persian version of SES reported acceptable face validity. The CVI and CVR values reported acceptable content validity for all SES items. The Cronbach's alpha coefficient (*α* = .86) and construct reliability (CR = 0.79) indicated good internal consistency between SES items. Based on the reported face, content, and construct validity, we can conclude that SES is a common trait between Spanish and Iranian parents.

The current study highlights that sharenting is a global trait that exists in individuals with Western and Persian cultural backgrounds. Because Persian culture encompasses various tribes and ethnicities, the fact that many of these parents are not open to sharenting, items 3, 7, 10, and 11 did not have sufficient factor loading. Due to these items' low factor loading did not align with the results of Romero‐Rodríguez et al. (2022).

In the present study, the results of convergent validity showed that there is a positive and significant relationship between sharing and constructs, such as social comparison, self‐control, implications, and social behaviors, but no significant relationship was found between social well being and sharing. It can be said in the present explanation, when parents today share pictures of their children in brand clothes and exchange fashion knowledge on social media they express their extended selves and create self‐representations for themselves and their children. Dress choices are one tool used to achieve the desired self‐representation on Instagram. Additionally, the images produce representations of childhood, circulating the symbolic meanings given to it (Kallioharju et al., 2023). The seeking of affirmation and positive reinforcement in sharenting is also a negative feature of the practice, which has been investigated. Parents have many reasons for using social media, such as finding social support online, being able to present themselves and their families, and feeling validated in the positive feedback they get through likes and comments (Ong et al., 2022; Ouvrein & Verswijvel, 2019).

### 4.1 Limitations, implications, and future research

Some limitations must be considered within the context of the current study. First, participants were not selected using random sampling; therefore, their responses may not provide an accurate reflection of the target population. In this study, the time parents spent on social media and the number of images they have shared of their children were not examined, which is a limitation. Moreover, self‐reports were used as the means of data collection in this study which introduced unique challenges to the nature of the research and biased perceptions a risk.

Given that the SES is a short scale, the evaluation of scores on this scale takes little time and effort, making it an efficacious research tool in larger‐scale studies to provide international comparative analysis of different cultures on several scales are employed simultaneously. The Persian SES can be employed on a national and international level to provide an accurate understanding of the impact of various cultural backgrounds on parental attitudes toward sharenting. However, it is imperative to note that the validity and reliability of this scale have only been approved within a sample of parents who live in Tehran. Future studies must examine SES validity and reliability in different Iranian populations (according to their education, the age range of 20–60, number of children, and the time parents spend on the Internet and social media).

Further studies are needed using the SES within international landscapes to support and extend SES validity to sample populations belonging to other cultures and the Persian culture more broadly. Scholars and parenting experts (REFS) advocate for parents to consider whether or not others can take advantage of their shared content before sharenting content about their children (e.g., photos, videos, and even health records) online. Parents should consider the negative consequences of what they share about their children on social media (Kopecky et al., 2020), and for this occur, widespread international guidelines, policy, and psychoeducation are needed to inform parents about the importance of maintaining their children's privacy, their children's right to consent, implications of sharing other children's information, and potential legal ramifications.

## 5 CONCLUSION

The present study sought to assess the psychometric properties of the SES based on the reported face, content, and construct validity. It was concluded that SES is a robust tool to use with Iranian parents. Implications of the research provide room to conduct further studies that examine the validity of SES in Eastern (Asian) and Western cultures. Intercultural studies afford researchers the chance to compare data between countries, and potentially providing culturally responsive tools and resources to Iranian parents.

## AUTHOR CONTRIBUTIONS

*Study designed; data collection; data analyses; writing draft*: Faezeh Peimanpak, Abbas Abdollahi, Kelly A. Allen, Feruza Abulkosimovna Rakhmatova, and Alaa Aladini. Shadia Hamoud Alshahrani and Jocelyn Brewer reviewed the manuscript, editing the manuscript.

## CONSENT TO PARTICIPATE

Informed consent was obtained from all individual participants included in the study.

## INFORMED CONSENT

Consent forms were signed from the participants

## CONFLICT OF INTEREST STATEMENT

The authors have no conflicts of interest to disclose.

### PEER REVIEW

The peer review history for this article is available at https://publons.com/publon/10.1002/brb3.3300.

## Data Availability

Data is available on Figshare repository at https://doi.org/10.6084/m9.figshare.21740597.
